# Korean American Women's Experiences with Smoking and Factors Associated with Their Quit Intentions

**DOI:** 10.1155/2013/796570

**Published:** 2013-08-24

**Authors:** Sun S. Kim, Seongho Kim, Gregory Seward, Lisa Fortuna, Sherry A. McKee

**Affiliations:** ^1^Department of Psychiatry, University of Massachusetts Medical School, Worcester, MA 01652, USA; ^2^Department of Social Welfare, Korean Bible University, Seoul 139-791, Republic of Korea; ^3^Department of Psychiatry, School of Medicine, Yale University, New Haven, CT 06511, USA

## Abstract

This study explored Korean American women's experiences with smoking and tested the theory of planned behavior to identify factors associated with their intentions to quit smoking. It employed a mixed-methods research design, using qualitative and quantitative data. Participants were recruited via a combination of random (*N* = 49) and convenience (*N* = 45) sampling techniques. Women in this study initiated smoking at age of 23 on average, and nearly half smoked at indoor houses. They initiated smoking out of curiosity about the effect and belief that smoking would relieve their stress. Reasons for continued smoking were (a) to avoid nicotine withdrawal symptoms, (b) to cope with life stressors, including acculturative stress, and (c) to fulfill one's destiny as a lifetime smoker. Many attempted to quit due to health issues and pregnancy. Fear of disclosure and limited English proficiency were found to be major barriers to seeking help for quitting. Past-year quit attempt(s), attitudes toward quitting, and perceived family norm favoring quitting explained 25% of the variance in intentions to quit smoking (*F*_[3,90]_ = 11.58, *P* < 0.001). Findings suggest that gender- and culture-specific intervention strategies are needed to assist Korean American women in smoking cessation.

## 1. Introduction

Tobacco use is widely recognized as the most preventable cause of illness and death in the United States (US). In 2010, an estimated 17.3% of US women were current smokers as compared to 21.5% of US men, supporting the historical difference seen in prevalence rates [1]. Of the US population, Asian Americans present the most striking gender difference in smoking rates [2–5]. While Asian men of some ethnic subgroups smoke at higher rates than the general US population, their female counterparts reportedly smoke at the lowest rate (4.3%) of all racial and ethnic groups [1]. Similar to what has been found among Asian men, however, smoking rates among Asian women vary across ethnic subgroups [2–5] and by acculturation level [5–7]. In addition, among certain segments of this population smoking rates have been on the rise. For example, the rates of smoking among Korean American women in California have been steadily increasing from 8% to 21% in the past decade [8–10]. 

Six tobacco research articles [5–7, 11–13] have been published to date that have any information on Korean American women, but findings are limited to reports on correlates of smoking and number of quit attempts. Korean American women who were not married were found to have more than 3 times the odds of being smokers as compared to married Korean American women [11]. Acculturation level showed a high correlation with smoking status [5–7]. In the study by Song and colleagues [7], the rate of current smoking was relatively high at 17.1% for the most culturally assimilated Korean American women, whereas it was low at 3.3% for the least acculturated. However, this difference might be related to a high rate of underreport among less-acculturated women as compared to the more acculturated. In California, 58% of Korean American women who smoked made a quit attempt within the past year, and the number of these attempts was about 2.9 on average [12]. Smoking Korean women were more likely to be exposed to secondhand smoke at home than nonsmoking Korean women [13]. 

Men in Korea typically become regular smokers during mandatory military service and continue to smoke, adhering to the cultural values of collectivism and conformity [14, 15]. In contrast, there is a strong cultural taboo against smoking by women in Korea. Reflecting the distinctive gender-based sociocultural norm of smoking, a recent population-based survey [16] in Korea showed a substantial underreport of smoking among women as compared to men when self-reported smoking rates were compared with urinary cotinine levels. Of 1,620 cotinine-verified smokers, 12.1% of men and 58.9% of women classified themselves as nonsmokers [16]. Korean American women may also underreport their use, particularly among those who are Korean-culture oriented. Given this, smoking prevalence in Korean American women may be much higher than what has been reported. 

None of the existing studies [5–7, 11–13] provided information on factors predicting quit intentions in Korean American women. Such information may help health workers be better equipped to address increasing smoking prevalence rates in this ethnic subgroup. To this end, we examined psychosocial variables that might explain Korean American women's quit intentions, using the theory of planned behavior (TPB) [17, 18]. A positive association of alcohol use with smoking has been found among Korean American men [19, 20] and Korean American college students [21]. We also examined in this study whether alcohol use would be a correlate of smoking in Korean American women. In addition, we explored their experiences with smoking and quitting, conducting in-depth interviews. We wanted to know why Korean American women started smoking, why they continued to smoke, what would motivate them to quit smoking, and what hindered them from seeking help for quitting.

The TPB posits that the more favorable attitudes, the more socially compelling norms, and the greater perceived behavioral control, the stronger will be an individual's intention to perform the behavior in question [17, 18]. The theory also proposes that the intention determines actual performance of the behavior. In this theory, the concept perceived behavioral control was borrowed from Bandura's [22] self-efficacy. Thus, TPB researchers (e.g., [23–26]) often use self-efficacy instead of perceived behavioral control, and a meta-analysis [27] revealed that self-efficacy had greater predictive power of behavioral intention and actual behavior than did perceived behavioral control. We also used self-efficacy instead of perceived behavioral control in this study. Based on the theoretical proposition of the TPB, Korean American women who had more positive attitudes and fewer negative attitudes toward quitting, stronger social norms favoring quitting, and greater self-efficacy in quitting would have greater intentions to quit smoking. 

## 2. Method

### 2.1. Design

This study employed a mixed-methods research design and is part of a larger telephone survey study conducted with a nationwide sample of Korean Americans. Findings pertaining to men were reported elsewhere [28]. Within the ethnic group, women were far less likely than men to disclose their smoking status, and, hence, we used multiple sampling strategies to recruit Korean American women. Given the paucity of published tobacco studies on this ethnic subgroup, we also conducted in-depth interviews with women only. The study was approved by the institutional review board of the first author's university. Verbal consent was obtained from each participant prior to the interview. Those who completed the telephone survey received a $40 gift card by mail. 

### 2.2. Participants

Participants were restricted to daily smokers because we wanted to gather information that could be adapted in the development of a smoking cessation program for Korean Americans. To be included in the study, they were required to (a) self-identify as Korean, (b) be able to speak English or Korean, (c) be of age of 18 or older, and (d) have smoked at least one cigarette a day for the past six months. 

### 2.3. Data Collection

The survey was conducted between October 2008 and September 2010. A list of households with a Korean surname such as “Kim” and “Lee” was created from an online telephone directory (http://www.infospace.com/). The households were identified from the top 100 Korean populated cities in 27 states based on the 2000 US Census [29]. Households were selected from the list with an identification number that was randomly generated. This sampling technique has been widely adopted in population-based studies of Korean Americans (e.g., [19, 20, 30]). Selected households were called up to seven times at various times of a day and days of a week, including evenings and weekends. Those who answered the call were inquired about ethnicity of the household and the presence of any adult smokers. We interviewed only one per household and a woman over a man if the household had both male and female smokers. If the smoker was not present at the time of the call, permission was obtained to call the number again and information was sought about the best time to contact the smoker. 

A respondent-driven sampling (RDS) technique was implemented for women only. The RDS is an innovative adaptation of chain-referral network sampling that provides peer-driven access to hard-to-reach subpopulations while reducing sampling biases associated with conventional snowball sampling [31, 32]. When an interview with a randomly selected smoker was completed, we asked the person to pass out our contact information to Korean women whose daily smoking status was certain to them. We also announced the study on the largest online community of Korean American women (http://www.missyusa.com/), calling female smokers for participation in a research study. 

Three bilingual Korean Americans (two female doctorates and one male doctoral student) conducted the survey in English or Korean depending on the preference of the person being interviewed. After the completion of the survey that took about 30 minutes on average, we further invited women who were randomly identified for an in-depth interview. This additional interview took 25 minutes on average. 

### 2.4. Measures

All measures but one described next had been used with Korean Americans in previous studies [23] and their psychometric properties have been reported elsewhere [33–35]. The measure “Perceived Risks and Benefits Questionnaire” [36] was used for the first time with Korean Americans. We developed a Korean version through a rigorous process of cross-cultural validation, including translations and back translations and pilot tests with Korean American smokers. 

#### 2.4.1. Sociodemographic Data

Demographic information was gathered in the following areas: age, marital status, education, annual family income, employment status, and health insurance coverage. 

#### 2.4.2. Acculturation

A brief form of the Suinn-Lew Asian Self-Identity Acculturation Scale [37] was used. The brief form has five question items (language spoken, language preferred, language read, childhood friends, and cultural identity) that had the highest item-to-total correlations among 21 items in its full version [38]. The Score for each item ranges from “1 = *very Korean-culture oriented*” to “5 = *very American-culture oriented,*” and the scale score is the mean score of the five items. 

#### 2.4.3. Alcohol Problem

The Alcohol Use Disorders Identification Test [39] was used. It was developed by the World Health Organization to be used as a simple method of screening for hazardous and harmful alcohol drinking. The measure consists of 10 items, and each item is scored from “0” (e.g., *never*) to “4” (e.g., *daily or almost daily*). The scale score is the sum of the scores of the 10 items. As recommended, a cutoff score of “8” was used to indicate alcohol problems. 

#### 2.4.4. Smoking and Quitting

The following information was obtained: the age at smoking onset, smoking at home and at work, number of cigarettes smoked per day on average, quit attempts that lasted at least 24 hours in the past year, and the number of the attempts and use of any smoking cessation aids including medications. 

#### 2.4.5. Nicotine Dependence

The Fagerström Test for Nicotine Dependence [40] consists of six items: four dichotomous and two multiresponse (0–3) items. The scale score is the sum of the scores of the six items, ranging from 0 to 10. Higher scores indicate higher dependence on nicotine. 

#### 2.4.6. Stage of Behavior Change for Quitting

Two dichotomous questions were used to assess stages of readiness for quitting [41]: (1) “Do you intend to quit smoking within the next six months?” and (2) “Do you have a plan to quit within the next month?” Based on answers to these two questions, participants were placed in (1) precontemplation stage if they had no intention to quit smoking within the next six months, (2) contemplation stage if they had an intention within the next six months but had no plan to quit within the next month, or (3) preparation stage if they had a plan to quit smoking within the next month.

#### 2.4.7. Attitudes toward Quitting

This variable was assessed using an indirect measure, the Perceived Risks and Benefits Questionnaire [36]. It consists of 18 items for perceived risks of quitting (negative attitudes: “I will be less able to concentrate” and “I will miss the taste of cigarettes”) and 22 items for perceived benefits of quitting (positive attitudes: “I will smell cleaner” and “I will feel proud that I was able to quit”). Items are rated on a 7-point Likert-type scale (“1” *= no chance at all, *“7” *= certain to happen*). The mean score is the scale score for each subscale. 

#### 2.4.8. Perceived Social Norms toward Smoking

This variable was assessed using a measure of two items regarding normative beliefs (e.g., “I believe that my family or my friends want me to quit smoking”) and motivation to comply (e.g., “I am willing to comply with the belief”) [42]. The score for each item ranges from “−3” (*strongly disagree*) to “+3” (*strongly agree*), and the scale score is the sum of the scores of the two items. Smokers are often conflicted by the discrepancy between perceived social norms for family and for friends, many of whom also smoke. Thus, scores of the two referent groups were not combined. 

#### 2.4.9. Self-Efficacy

This variable was assessed using a 10-item, 5-point Likert-type scale that rates confidence in one's ability to resist smoking temptation in ten high-risk situations (e.g., “When I feel tense or anxious” and “When I wake up in the morning”) [35]. The measure was adapted from the Smoking Abstinence Self-Efficacy Scale [43]. The score for each item ranges from “1” (*completely unconfident*) to “5” (*completely confident*), and the scale score is the sum of the scores of the ten items. 

#### 2.4.10. Intentions to Quit Smoking

They were assessed using a 4-item, 7-point Likert-type scale (e.g., “I intend to quit smoking within the next two weeks” and “I will make an effort to quit smoking within the next two weeks”) [23]. The score for each item ranges from “−3 = *most unlikely*” to “+3 = *most likely*.” The scale score is the mean of the scores of the four items and higher scores indicate more intentions to quit smoking.

### 2.5. Qualitative Data

We initially planned to have a focus group interview, but most women declined to participate in such a group interview due to the fear that their smoking status could be known to everyone in their small Korean community. As an alternative, individual interviews were conducted with those who were randomly selected from the online telephone directory. The following four open-ended questions were used: (a) tell me how you started smoking, (b) tell me why you continued to smoke, (c) tell me what made you want to quit, and (d) tell me whether you have perceived or experienced any barriers to seeking help for quitting. 

### 2.6. Data Analysis

Quantitative data were analyzed using the SPSS version 20.0. Descriptive statistics were used to characterize the sample with respect to sociodemographic data, acculturation, alcohol problems, age at smoking initiation, nicotine dependence, quit attempt(s) in the past year, and the TPB variables (attitudes, perceived social norms, self-efficacy, and quit intentions). All variables were assessed for multicollinearity, skewness, and violation of normal distribution. Chi square tests for categorical variables and two-sample *t*-tests for continuous variables were performed to compare demographics and key study variables between two samples: random and convenience. Pearson's or Spearman's rho correlation coefficients were obtained to identify correlates of quit intentions, and stepwise forward linear regression analyses were performed to find significant predictors of quit intentions. Demographics and smoking behavior were entered first and then TPB variables to examine whether the variables had additional explanatory power of the variance in quit intentions. 

Two bilingual Korean doctorates independently analyzed qualitative data using a constant comparison method [44]. They independently identified key words from participants' narratives and coded them into themes. They then grouped the themes into categories: smoking initiation, smoking continuation, quit attempts, and actual and perceived barriers to seeking help for quitting. The two analyzers compared the themes in each category and discussed until they resolved discrepancies. Member checking [45] was also implemented by asking questions in subsequent interviews to validate emerging themes. 

## 3. Results

The sample comprised 94 women (Table 1). The random sample was more likely to be older (*t*_92_ = 5.14, *P* < 0.001) and less educated (*t*_92_ = −2.03, *P* = 0.05) than the convenience sample. The random sample was more likely to be interviewed in English than the convenience sample (22.4% versus 2.2%; *χ* ^2^[1,  *N* = 94] = 8.62, *P* < 0.01). The random sample had greater intentions to quit smoking within the next two weeks than the convenience sample (*t*_92_ = 2.46, *P* = 0.02). However, stage of behavior change for quitting did not differ between the two. Although marginally significant (*P* = 0.06), the random sample initiated smoking later than the convenience sample. 

The mean age of all participants was 46.6 with a range of 18 to 88. Exactly half were married, and the vast majority of them (82.9%) were married to husband who also smoked. Individuals (12.8%) interviewed in English started smoking earlier (*t*_92_ = 2.08, *P* = 0.04) than those (87.2%) interviewed in Korean. Almost half of the participants (48.9%) smoked more than 10 cigarettes a day, and one third (33.0%) smoked within 30 minutes after waking. Nearly 50% of the women smoked indoors, and the vast majority of them had other family members who also smoked indoors. Slightly more than half (52.1%) attempted to quit smoking in the last year, and of these women, approximately one third (33.0%) used cessation medications and three had cessation counseling (data not shown). 

## 4. Factors Associated with Quit Intentions

Regression analyses yielded similar results in factors associated with quit intentions between the two samples. Thus, data were combined, and results are presented in Table 2. The presence of past-year quit attempts was a significant factor of quit intentions, and this variable alone explained 12% of the variance. Among the TPB variables, negative attitudes (i.e., perceived risks of quitting) and perceived family norm were significant factors of quit intentions even after controlling for past-year quit attempt(s). The TPB variables explained an additional 13% of the variance. Although perceived friend norm was a significant factor of the intentions, it was no longer significant when perceived family norm was controlled for. 

## 5. Experiences with Smoking and Quitting

Of the 49 women who were randomly selected, 31 (63.3%) agreed to do an in-depth interview. Themes were identified from statements provided in response to the four open-ended questions. They were grouped into four categories: (a) reasons for smoking initiation, (b) reasons for continued smoking, (c) reasons for quitting, and (d) barriers to seeking help for quitting.  Table 3 is the frequency chart of themes that were identified for each of the four categories. The numbers indicate how many times each theme was addressed by the women who participated in the in-depth interview. 

### 5.1. Reasons for Smoking Initiation

Curiosity about the effect of smoking was the theme most frequently mentioned for smoking initiation. Women who endorsed stressful immigrant life as the reason mostly initiated smoking in middle age. Some stated that they started smoking to resist against the gender-based cultural norm of smoking in Korea. Some women initiated smoking affected by more than one factor as the case shown next:

*I started not long after coming here [the United States]. One day I was so stressed out and decided to smoke [a cigarette]. I was very curious about its effect because my husband smoked a lot around me. That was how I got hooked on this. Now, I don't care about my health. Smoking is the only pleasure I have now. *

In addition, four reported Korean culture-specific circumstances that led them to smoking. A 74-year-old woman stated that she started smoking at age 34 to relieve severe morning sickness during pregnancy. Her mother-in-law was the one who strongly encouraged her to smoke at the time. She smoked throughout the pregnancy and afterward. Another woman aged 85 told that she became addicted to smoking by lighting cigarettes for her father-in-law at his grave. Adhering to Confucianism in old Korea, offspring, particularly the firstborn son and his wife, were required to live close by the grave of a deceased parent for three years from the burial. Every day, they had to serve the deceased parent three meals. They also lit cigarettes and left it next to the meal if the deceased was a smoker. They believed that the spirit of the deceased parent would eat the food and smoke the cigarette. 

### 5.2. Reasons for Continued Smoking

Avoidance of withdrawal symptoms was the most frequently mentioned reason for continued smoking. Many seemed to realize that they were not able to quit smoking after experiencing severe withdrawal symptoms. They stated how horrible the experience was. “I am never going to try it [quitting] again. I suffered too much. It was unbearable. I'd rather die [than quitting],” said one woman. 

Smoking being a coping strategy for life stressors was the next most frequently addressed reason for continued smoking. Many mentioned that smoking is the only pleasure they had, and that, as an immigrant, living in this country without smoking was unbearable. Another woman aged 62 stated her struggle with smoking as follows:

*It's hard to live without smoking especially since my son died. I wonder whether he could have been alive had we not come here [the United States]. One day all of a sudden he collapsed and was not able to be brought back. He worked hard without any days off. I know smoking is not good for health. Yet, I can't help it. I tend to smoke more whenever I think of him. *

She lost her second son who died of heart attack about two years ago. Since his death, according to her, her husband, her first son, and she had quit smoking for about a month but all relapsed, smoking more than before the quitting. Similar to her, many stated that they could not help but smoking because it was the only way to cope with their life stressors.

“Smoking is my destiny” was another frequent sentiment shared, particularly among older women as a reason for continued smoking. One woman aged 88 said: “I am too old to quit. I know my family wants me to quit but I will smoke until I die.” Another aged 76 said: “My family just wants me not to smoke too many. They know that I cannot quit. This is my destiny.” In addition, a good number of women mentioned having coaddiction with alcohol was the reason for continued smoking. 

Irrespective of the reasons for continued smoking, many stated that their life could have been much better had they not initiated. Some described in detail how smoking had changed their lives. The woman who initiated smoking due to morning sickness in pregnancy poignantly described her life situation as follows:

*Every time I light a cigarette I curse the day that I ever put my hands on [cigarette]. Due to this [smoking], I cannot go anywhere. I now live in a cage. Every Korean in this community knows that I had the surgery. Had they seen me smoking, they would think I am crazy. They would gossip me. So, I don't smoke outside. I just stay here.*

About two years ago, she was diagnosed with a stomach cancer and subsequently had an operation. She had quit for about six months and then relapsed by smoking cigarette butts that her friends left in an ash tray when they came to see her. She wished that she could visit friends in Korea before she dies. Yet, she mentioned that she might not be able to do that because of her high nicotine dependence. Not being able to smoke for 13 hours while riding in a plane made her change her mind. She attempted to use nicotine patches but her craving for cigarettes was still unbearable. Similar to this woman, the vast majority of the women living in a Korean-populated area such as Los Angeles, CA, USA, and New York city, NY, USA, reported that they did not smoke outside of their house due to the fear of being caught while smoking by Korean neighbors.

### 5.3. Reasons for Quitting

Health concern was one of the major factors motivating Korean women to quit smoking. For example, “I know I have to quit because of the problem [asthma] … yet, it is not easy because my husband also smokes,” said one woman aged 50. Many indeed attempted to quit smoking after the diagnosis of a serious health problem and then realized that quitting was much harder than what they had expected. A woman aged 47 described her daily struggle with quitting as follows:

*About a year ago I learned that I have emphysema. I was hospitalized and at the time, I had not smoked for about three months. Yet, one day I was very angry from work. I got one [cigarette] from my brother and smoked the whole cigarette…. Since then, I smoke about 10 cigarettes a day. I tried to quit many times, almost every day for a whole month…. I even called the quit line but [it was] not helpful. Nothing seems to work. So I don't try it [quitting] any more. This is why I now climb a mountain every weekend … to slow down the damage that I am causing to the lung by smoking. *

Pregnancy was the other major factor motivating Korean women to quit. Many reported that they indeed had been abstinent in pregnancy although they relapsed not long after giving birth. One woman aged 38 said the following:

*I stopped [smoking] because I could feel the baby was moving a lot whenever I smoked. It seemed like the baby was not feeling comfortable. So I stopped [smoking] from the 7th month [of pregnancy].*

### 5.4. Barriers to Seeking Help for Quitting

More than half of the women interviewed in depth stated that they had never thought of seeking help for quitting. Particularly, young women in their 20s and 30s tended to believe that they could quit on their own, and thus, there would be no need to seek help. However, those women who had made quit attempts seemed to be in agreement that quitting was much harder than what they had expected. Among the barriers identified, the fear of disclosing their smoking status to others was most frequently reported. One woman aged 59 stated the following:

*One time a [Korean] community center distributed free patches. Only smokers were allowed to get them with an id card. So going there is like telling people that I smoke. Yet, I don't want to do this. Some who work there could be my neighbors. *

In addition, limited English proficiency, financial constraint, and time constraint were identified as barriers to seeking help.

## 6. Discussion

To our knowledge, this is the first study investigating factors associated with Korean American women's intentions to quit smoking. Among the key study variables, past-year quit attempts, negative attitudes toward quitting (i.e., perceived risks of quitting), and perceived family norm favoring quitting explained 25% of the variance in Korean American women's quit intentions. Findings pertaining to demographic characteristics and a history of quitting were similar to those reported in previous studies [11–13] with Korean American women. Only 41% of the women in the random sample were married, and the rate is much lower than what has been reported for the general Korean female population in the United States [5–13]. The proportion of Korean female smokers who made past-year quit attempts and the average number of the attempts found in this study were similar to those in the other study [12]. 

Among smoking-related variables, past-year quit attempt was a significant factor of quit intentions in Korean American women. It has been reported that former smokers in the general US population made 4.7 quit attempts on average before they succeeded in quitting [46]. Another important aspect of quit history is the time interval since the last quit attempt, such that smokers who made a more recent attempt (e.g., quit attempts made within the past year) were more likely to try again in comparison to smokers whose last attempt was longer ago [47]. Unlike the finding with Korean American men [23], nicotine dependence was not a significant factor of the intentions. This might be related in part to the recent finding that the Fagerström Test for Nicotine Dependence is not a valid measure of nicotine dependence for Korean American women although it has shown sound psychometric functions for Korean American men [33]. Most women in this study seemed to have a high level of nicotine dependence given that one third of them smoked the first cigarette within 30 minutes of waking and many had several serious quit attempts in the last year. In addition, a good number of the women mentioned that they continued smoking in order to avoid nicotine withdrawal symptoms. 

Similar to what others found in the general US female population [36, 48], negative attitudes toward quitting (i.e., perceived risks of quitting) were associated with quit intentions among Korean American women, whereas positive attitudes toward quitting (i.e., perceived benefits of quitting) were not. It was reported that women were more motivated to quit when they perceived fewer risks of quitting, whereas men were more motivated to do so when they perceived more benefits of quitting [36]. Perceived family norm favoring quitting showed a significant association with quit intentions even after adjustment for other covariates, which is in concert with a previous study with Korean American men [23]. Similarly, Vietnamese male smokers in California who reported smoking causing family conflicts were more willing to quit than those who did not [49]. Self-efficacy was not a significant correlate of quit intentions. This finding is similar to the finding from a previous study with Korean American men [23]. Self-efficacy may lack explanatory power when retrospective and prospective estimates are used for its assessment, because the variable is not a static entity and changes over time [50]. Instead, ecological momentary assessment was recommended for use because this assessment method monitors daily self-efficacy as it unfolds in the natural environment. 

Korean women's experiences with smoking and quitting seemed to be largely different from Korean men's in previous studies [11, 51]. Factors such as cultural conformity, cigarettes being a social medium, and smokers being a male gender identity that were found in relation to Korean men's smoking initiation and continuation [11] were not endorsed by Korean women in this study. Instead, curiosity, avoidance of nicotine withdrawal symptoms, and stressful immigrant life were identified as the main reasons for Korean women's smoking initiation and continuation. Findings pertaining to pregnancy and fear of disclosure were also unique to women. Despite these differences, Korean American female and male smokers shared similarities in their struggle with nicotine withdrawal symptoms and willingness to quit due to health concerns [51]. 

Women who lived in a Korean dense area reported smoking secretly only inside the house due to the fear of being caught while smoking by a Korean neighbor and being subjected to gossip. A similar finding was reported with female smokers in Cape Town, South Africa [52], who reported smoking secretly in the restroom. Almost half of Korean American women in this study lived with other family members who also smoked indoors. These findings were supportive of the finding that Korean women who smoke were more likely to be exposed to secondhand smoke at home as compared to nonsmoking Korean women [15]. Given that Korean women who smoke are more likely to be unmarried or married to a smoker rather than married to a nonsmoker, the household may not have an indoor smoking ban. 

Similar to the finding with Hmong American female smokers [53], many women in this study reported difficulty seeking treatment due to the fear of disclosure. This may be one of the underlying reasons for a low participation rate of Asian American women in community-based smoking cessation programs (e.g., [54–56]) even after their low smoking rate has been taken into consideration. There is an urgent need to examine the feasibility and effectiveness of cessation interventions for Asian American women that are different from the conventional approach. Asian American women may feel less threatened if smoking cessation interventions are delivered via online quit programs or telephone counseling which can be accessed at home without revealing their smoking status to others in the community. California currently provides free cessation counseling via a quitline service with an Asian language option [57], and other Asian-populated states such as New York should expand the language option to Asian languages. 

The present study has several limitations. First, we recruited the majority of the women from an online telephone directory that listed only landline telephone numbers. Thus, findings from the study might be limited to middle- and old-aged women [58]. This may have caused in part the differences found between the random and convenience samples. Second, women in this study were all daily smokers, and findings may not be applicable to intermittent and social smokers. Third, the level of nicotine dependence of the women in this study could not be assessed accurately because of the problems inherent in the Fagerström Test for Nicotine Dependence [33]. Fourth, not all women of the random sample participated in the in-depth interview. 

Despite the limitations stated earlier, the present study was the first study with Korean American women, providing detailed personal accounts of smoking and quitting behavior. Findings underscore the importance of gender- and culture-specific approaches to tobacco dependence treatment for Korean American women. Particularly, the treatment should be focused on enhancing alternative coping skills to deal with life stressors and managing effectively nicotine withdrawal symptoms. Furthermore, given the similar sociocultural proscription against smoking by women in most Asian countries, Asian women irrespective of ethnicity may share some of the experiences that Korean women had. More studies are urgently needed to compare similarities and differences in experiences with smoking and quitting across ethnic subgroups of Asian American women. 

## Figures and Tables

**Table 1:** Demographics and study variables by sample.

Variable	Random (*N* = 49)	Convenience (*N* = 45)	All (*N* = 94)
	Mean ± SD/*N* (%)	Mean ± SD/*N* (%)	Mean ± SD/*N* (%)
Age∗∗∗	53.7 ± 15.7	38.7 ± 12.3	46.6 ± 16.0
Marital status (= married)	20 (40.8%)	27 (60.0%)	47 (50.0%)
Years of education∗	11.8 ± 4.2	13.5 ± 3.6	12.6 ± 4.0
Family income			
<$20,000	14 (28.6%)	8 (17.8%)	22 (23.4%)
$20,000–$59,999	13 (26.5%)	20 (44.4%)	32 (35.1%)
$60,000–$99,999	9 (18.4%)	13 (28.9%)	22 (23.4%)
≥$100,000	8 (16.3%)	4 (8.9%)	12 (12.8%)
Refused	5 (10.2%)	0 (0.0%)	5 (5.3%)
Employment (= yes)	33 (67.3%)	33 (73.3%)	66 (70.2%)
Health insurance (= yes)	34 (69.4%)	25 (55.6%)	59 (62.8%)
Acculturation (1–5)	2.1 ± 1.0	1.9 ± 0.4	2.0 ± 0.8
Alcohol problems (= yes)	7 (14.3%)	11 (24.4%)	18 (19.1%)
Age at smoking onset^†^	24.1 ± 8.8	21.3 ± 4.9	22.8 ± 7.3
Nicotine dependence (1–10)	4.0 ± 2.6	3.7 ± 2.0	3.8 ± 2.3
Smoking at home (= yes)	21 (42.9%)	24 (53.3)	45 (47.9%)
Smoking at indoor work place (= yes)^a^	4 (12.1%)	3 (9.1%)	7 (10.9%)
Past-year quit attempts (= yes)	26 (53.1%)	23 (51.1%)	49 (52.1%)
Number of past-year quit attempts (= yes)^b^	3.0 ± 2.2	3.2 ± 2.6	3.1 ± 2.4
Stage of quitting			
Precontemplation	20 (40.8%)	19 (42.2%)	39 (41.5%)
Contemplation	10 (20.4%)	16 (35.6%)	26 (27.7%)
Preparation	19 (38.8%)	10 (22.2%)	29 (30.8%)
Positive attitudes toward quitting (1–7)	4.2 ± 1.3	4.5 ± 1.2	4.3 ± 1.3
Negative attitudes toward quitting (1–7)	5.3 ± 1.0	5.6 ± 0.9	5.4 ± 1.0
Perceived family norm for quitting (−6–+6)	3.2 ± 2.6	3.1 ± 2.6	3.2 ± 2.6
Perceived friend norm for quitting (−6–+6)	1.3 ± 2.6	1.2 ± 2.8	1.2 ± 2.7
Perceived self-efficacy in quitting (10–50)	28.2 ± 8.0	27.5 ± 9.5	27.9 ± 8.7
Quit Intentions (−3–+3)∗	−0.1 ± 2.3	−1.2 ± 2.2	−0.6 ± 2.3

^a^Assessed only with those (*n* = 66) who worked at an indoor office, ^b^assessed only with those (*n* = 49) who made a serious quit attempt in the past year, ^†^ *P* < 0.10, ∗*P* < 0.05, and ∗∗∗*P* < 0.001.

**Table 2:** Factors associated with quit intentions in Korean American women (*N* = 94).

Stage	Variable	*B *	Std. error	Beta	*P* value	Adjusted *R* ^2^
Step 1						0.12
	Past-year quit attempt(s)	1.65	0.45	0.36	0.000	
Step 2						0.25
	Past-year quit attempt(s)	1.03	0.44	0.22	0.021	
	Negative attitudes toward quitting	−0.36	0.17	−0.19	0.039	
	Perceived family norm for quitting	0.34	0.08	0.38	0.000	

*B*: Unstandardized coefficient, std.: standard, and beta: standardized coefficient.

**Table 3:** Frequency chart of themes per category.

Themes	*N *
Smoking initiation	
Curiosity about the effect	17
Stressful immigrant life	9
Resistance to the gender-based social norm	4
Korean-specific cultural practice	4

Continued smoking	
Withdrawal symptoms	20
Life stressors	11
My destiny	6
Addiction to alcohol	6

Quitting	
Health concerns	28
Pregnancy	7

Barriers to seeking help for quitting	
Fear of disclosure	9
Limited English proficiency	8
Financial constraint	6
Time constraint	2
